# Meta-Analysis of Materials and Treatments Used in Contact Lenses: Implications for Lens Characteristics

**DOI:** 10.3390/ma18071445

**Published:** 2025-03-25

**Authors:** Ana Paula Oliveira, Clara Martinez-Perez

**Affiliations:** 1 Instituto Superior de Educação e Ciências de Lisboa (ISEC Lisboa), Alameda das Linhas de Torres, 179, 1750-142 Lisboa, Portugal; ana.oliveira@iseclisboa.pt; 2 Centro de Investigação, Desenvolvimento e Inovação em Turismo (CiTUR)—Polo Estoril, Avenida Condes de Barcelona, n. 808, 2769-510 Estoril, Portugal

**Keywords:** contact lenses, optical materials, lens coatings, lens characteristics, oxygen permeability

## Abstract

A meta-analysis was conducted to assess the evolution of, applications of, and recent advancements in materials and surface treatments for contact lenses. This study aimed to comprehensively synthesize the available data, focusing on innovations that enhance vision correction, comfort, and safety while emphasizing sustainability as a critical factor in future development. Registered with PROSPERO, this analysis adhered to the PRISMA and AMSTAR-2 guidelines. A systematic review of databases including PubMed, Web of Science, and Scopus was performed for studies published between 2019 and 2024, without language restrictions. Observational studies on optical materials and lens treatments were included, and a random-effects model was used to address the high heterogeneity among the included studies. From the nine studies that were analyzed, significant advancements were identified regarding the functional properties of materials and treatments. Key advancements included technologies like self-lubricating lenses that reduce friction, nanogels for prolonged therapeutic drug delivery, and coatings that minimize protein and lipid deposition, ensuring greater comfort and extended wearability. Additionally, innovations in biodegradable and eco-friendly materials underscore the industry’s commitment to reducing the environmental impact of contact lenses, addressing challenges related to lens disposal and recycling. These advancements highlight the potential of integrating functional improvements with sustainability, paving the way for more effective and environmentally responsible contact lenses.

## 1. Introduction

Contact lenses are thin, curved lenses that are placed directly on the surface of the eye for vision correction, cosmetic enhancement, or therapeutic purposes [1]. They have become a vital part of modern eye care, offering a practical and often superior alternative to traditional ophthalmic lenses. Contact lenses provide a broader field of vision, resist fogging, and are less impacted by adverse weather conditions, making them highly advantageous for use in sports and physical activities [1]. Cosmetic lenses, designed to alter the eye’s appearance, have also gained widespread popularity. Additionally, therapeutic lenses play a crucial role in delivering medications, protecting damaged corneas, and managing conditions such as keratoconus and dry eye syndrome [2,3,4].

The global prevalence of vision disorders, including myopia, hyperopia, astigmatism, and presbyopia, has fueled the rapid expansion of the contact lens market [1]. For example, they are prescribed for managing severe dry eye conditions in Stevens–Johnson syndrome, Sjögren’s syndrome, and chronic epithelial diseases, as well as for post-refractive surgery recovery [5]. According to the World Health Organization (WHO), approximately 2.2 billion people worldwide suffer from vision impairment [6,7,8], highlighting the urgent need for effective corrective solutions. In 2023, the global contact lens market was valued at approximately USD 18.6 billion and is projected to grow at a compound annual growth rate (CAGR) of 8.9% from 2024 to 2030 [9]. This growth reflects the increasing demand for innovative, comfortable, and multifunctional contact lenses.

The origins of contact lenses date back to the 16th century when Leonardo da Vinci conceptualized altering one’s visual perception by submerging the eye in water or using curved glass. In the 17th century, René Descartes expanded on these ideas with a similar vision correction concept [5,10]. The first practical contact lenses were developed in 1888 by German ophthalmologist Adolf Gaston Eugen Fick. Made from blown glass, these scleral lenses rested on a saline-filled space to protect the cornea. However, their impractical design and lack of comfort limited their use [10]. The 20th century witnessed significant advancements in contact lens materials, largely driven by the development of plastics. In the 1930s, polymethyl methacrylate (PMMA) lenses became the first durable and comfortable option. However, PMMA’s lack of oxygen permeability led to discomfort during extended wear [3,11,12]. The 1960s marked a turning point with the introduction of soft contact lenses made from hydrogel materials. These water-containing polymers allowed oxygen to pass through to the cornea, significantly improving the wearer’s comfort and the lens’ wearability [13,14,15]. The late 1990s ushered in silicone hydrogel lenses, which offered unprecedented oxygen permeability, addressing the limitations of earlier materials. These lenses became the foundation of modern contact lens technology and remain widely used today.

Advances in nanotechnology and biotechnology have revolutionized contact lenses, enabling the development of smart lenses. These innovative lenses are capable of [16,17,18,19,20]:

- Health monitoring: measuring intraocular pressure, glucose levels, or other health metrics;
- Drug delivery: the sustained release of medications directly to the eye;
- Augmented reality: providing visual overlays for enhanced navigation, training, or entertainment.

To overcome the limitations of the base materials and optimize lens performance, various surface treatments are employed. These treatments address key challenges such as hydration, protein deposition, and oxygen permeability [1,19,21]:

- Wettability enhancements: coatings and plasma treatments improve water retention, reducing dryness and increasing comfort;
- Anti-protein adsorption: specialized treatments minimize the buildup of proteins and lipids, maintaining lens clarity and reducing irritation risks;
- Oxygen permeability optimization: advanced technologies ensure sufficient oxygen flow to the cornea, preventing hypoxia and promoting corneal health;
- Therapeutic functionalities: coatings for sustained drug release convert lenses into therapeutic devices, delivering medications over extended periods;
- Self-lubricating surfaces: innovative coatings reduce the friction between the lens and eyelid, enhancing comfort and minimizing irritation.

The synergy between the materials and surface treatments employed plays a crucial role in defining the key attributes of contact lenses [22,23]. Optical transparency is essential, requiring materials and coatings that maintain clarity to ensure consistent, high-quality vision under various conditions. The water content and hydration are also significant factors; while a higher water content improves comfort, it must be carefully balanced with oxygen permeability to preserve corneal health. The durability and elasticity of the lens material are determined by its elastic modulus, which influences the fit and comfort, particularly for users with sensitive or irregular corneas. Biocompatibility is another critical aspect, as minimizing inflammatory responses and improving cell viability enhances both the safety and long-term wearability of contact lenses. Furthermore, their therapeutic potential is expanded by innovations in materials and surface treatments that enable targeted drug delivery, broadening the applications of lenses in medical fields.

This meta-analysis aims to evaluate the evolution of, applications of, and recent advancements in materials and surface treatments used for corrective eyewear, specifically focusing on contact lenses. The objective is to provide a comprehensive synthesis of the available data to understand how these innovations enhance vision correction, comfort, and safety, while also considering sustainability as a key factor in the development of future technologies. This study follows the PRISMA and AMSTAR-2 guidelines, ensuring transparency and reproducibility in the systematic review process.

In recent years, the integration of machine learning (ML) techniques into meta-analyses has gained increasing attention, particularly its use for automating data extraction, assessing bias, and enhancing predictive modelling [24,25,26]. Artificial neural networks and other ML algorithms can efficiently analyze large datasets and identify patterns that traditional methods may overlook [26]. In the context of contact lens materials, ML could help predict the performance of lenses based on their chemical composition, manufacturing techniques, and clinical outcomes. Although ML-enhanced meta-analyses are still an emerging field, they offer significant potential to refine data synthesis and provide deeper insights into material properties [27]. However, their complexity may introduce challenges in interpretability and reproducibility, potentially affecting adherence to frameworks like PRISMA and AMSTAR-2 [28,29]. Integrating ML into meta-analyses could not only improve their analytical precision but also open new possibilities for optimizing contact lens material selection and performance evaluation.

## 2. Materials and Methods

### 2.1. Eligibility Criteria

**Figure 1:**
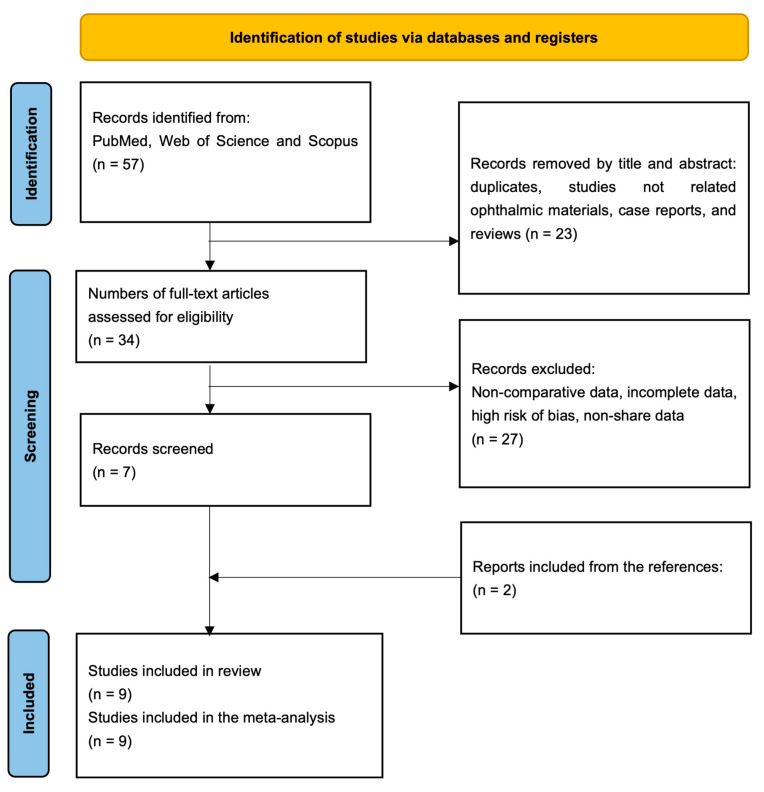
Flow diagram for study selection (preferred reporting items for systematic reviews and meta-analysis).

This meta-analysis was registered with PROSPERO (CRD42025641647) and conducted in accordance with PRISMA guidelines (see PRISMA 2020 Checklist Supplementary Materials S1) [30] and AMSTAR-2 standards [31] (Figure 1). The research question was framed using the PICOS framework, ensuring a structured approach. The analysis focused on observational studies investigating contact lenses fabricated from various materials, including hydrogels, silicone hydrogels, and nanocomposites. These materials were treated with advanced technologies such as biocompatibility-enhancing coatings and functional modifications. The interventions involved contact lenses designed for therapeutic or corrective purposes. These lenses incorporated innovative properties, such as sustained drug release, enhanced oxygen permeability, resistance to protein adsorption, and self-lubricating features.

The comparator group consisted of standard contact lenses without specialized treatments or the use of conventional technologies. This facilitated direct comparisons of their physical, chemical, and functional attributes. The primary outcomes assessed included optical transparency, water content, oxygen permeability, water contact angle, elastic modulus, drug release efficacy, and cell viability.

These outcomes were analyzed to evaluate the efficacy, durability, and biocompatibility of the materials, both in controlled experimental conditions and, where possible, in simulated real-world scenarios.

The meta-analysis exclusively included observational studies that quantitatively measured material properties under rigorously controlled laboratory conditions, ensuring reliability and reproducibility in the reported findings. This systematic approach provides a comprehensive evaluation of the advancements in contact lens materials and technologies.

### 2.2. Information Source

A comprehensive literature review was performed across multiple databases, including PubMed, Web of Science, and Scopus. The search focused on studies published within the past five years (2019–2024), ensuring the inclusion of recent and relevant research. A systematic and rigorous approach was adopted, with no restrictions on language to maximize the broadness of the review.

To enhance the thoroughness of the search, the bibliographic references of studies identified in the initial phase were meticulously examined. This secondary review aimed to uncover additional studies that might have been overlooked in the primary database search, ensuring a more exhaustive and inclusive analysis of the available literature.

### 2.3. Search Methods for Identification of Studies

The search strategy employed the following terms to query all trial records and databases: (“Eco-friendly material*” OR “Sustainable material*” OR “Biodegradable material*” OR “Recycled material*”) AND (“Contact lens*”) (see PubMed search strategy in Supplementary Materials S1). To ensure the integrity and accuracy of the study selection process, two independent reviewers evaluated the identified studies. Any discrepancies were resolved through discussion, with a consensus ultimately being reached on the inclusion of eligible studies.

Two reviewers independently reviewed and extracted data from the selected studies. Key characteristics of each study were recorded, including the study name, timeframe, geographical region, study type, sample size, and lens materials investigated. The primary variables compared across studies were the refractive index and transmittance of the optical materials and coatings.

The methodological quality and risk of bias were evaluated independently by two reviewers using the Cochrane Collaboration’s Risk of Bias tool, which was facilitated by Review Manager software (RevMan) (Version 5.4, Cochrane Collaboration, London, UK). This tool provides a structured assessment across five critical domains of bias: material selection, consistency and reproducibility, objectivity in evaluation, management of incomplete data, and quality of reporting. For each domain, pre-defined criteria were applied to categorize the risk as low, high, or unclear.

**Figure 2:**
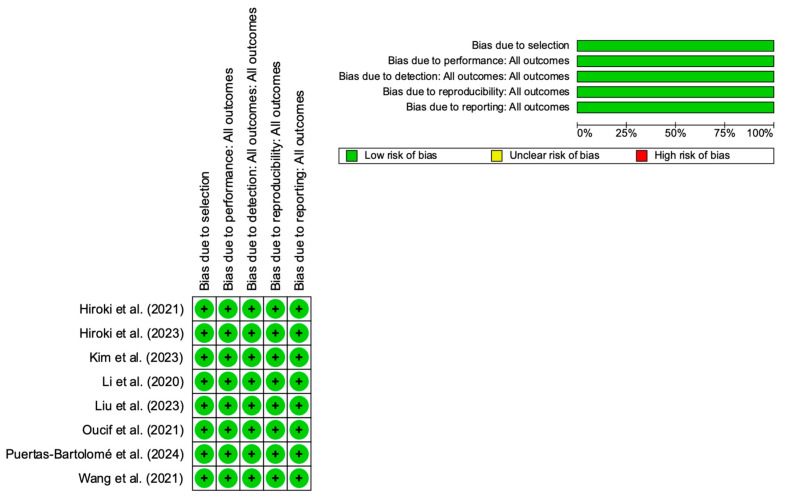
Risk of bias assessment [32,33,34,35,36,37,38,39].

The risk of bias for the included studies was assessed using the Cochrane Risk of Bias tool, covering five domains: selection bias, performance bias, detection bias, reproducibility bias, and reporting bias. Figure 2 provides a visual summary of the assessment. The top panel of the figure presents a bar chart summarizing the proportion of studies falling into each risk category across all domains. All included studies were classified as having a low risk of bias in all evaluated domains, as represented by the 100% green bars. The bottom panel displays the risk of bias for each individual study. Each row corresponds to a study, while each column represents a bias domain. Consistently, all studies show a low risk of bias across all assessed categories, as denoted by the green plus signs (+). The summarized results of this assessment are presented in Figure 2, while detailed justifications for each criterion are provided in Supplementary Materials S1 (risk of bias judgement). This systematic evaluation ensured a rigorous appraisal of study quality and reliability.

### 2.4. Assessment of Results

Mean differences (MDs) with corresponding 95% confidence intervals (CI) were calculated for continuous variables measured on the same scale. For variables assessed on differing scales, standardized mean differences (SMDs) were utilized to enable comparisons across studies. Heterogeneity among studies was evaluated using the I^2^ statistic, with values categorized as having low (<25%), moderate (25–50%), or high (>50%) heterogeneity. Given the substantial heterogeneity observed, random-effects models were employed rather than fixed-effects models to account for variability across studies. All statistical analyses were conducted using JASP statistical software (Version 0.19.1, JASP Team, Amsterdam, The Netherlands), which provides an open-source and user-friendly platform for advanced statistical analysis, ensuring the transparency and reproducibility of the results.

### 2.5. Publication Bias

A funnel plot analysis was conducted using JASP software (Version 0.19.1, JASP Team, Amsterdam, The Netherlands) to evaluate the potential presence of publication bias. Asymmetry in the funnel plot can suggest the existence of publication bias, often linked to the underrepresentation of smaller studies with non-significant or inconclusive findings. This analysis provides a visual and statistical means of assessing the robustness of the included studies and identifying potential biases in the reported outcomes.

### 2.6. Additional Analyses

Subgroup analyses were conducted to investigate variations in results across different factors, including transparency, water content, oxygen permeability, water contact angle, and cell viability. Additionally, sensitivity analyses were performed to evaluate the robustness of the findings. This involved systematically removing the most influential studies within each subgroup to determine the impact of individual studies on the overall conclusions. These approaches ensured a comprehensive assessment of potential variability and strengthened the reliability of the results.

## 3. Results

### 3.1. Study Selection

This study’s selection process followed the PRISMA guidelines, ensuring a transparent and systematic approach. The initial search identified 57 articles from PubMed, Web of Science, and Scopus (Figure 1). After we removed 23 duplicates, the full texts of the remaining 34 articles were carefully reviewed. Of these, 27 studies were excluded due to failure to meet the inclusion criteria, a lack of comparable variables, a high risk of bias, or incomplete data.

Ultimately, seven studies met the eligibility requirements and were included in the analysis. Additionally, two more studies were identified through reference screening of the included articles, resulting in a final selection of nine studies, all of which were incorporated into the systematic review and meta-analysis. The PRISMA flowchart shown in Figure 1 visually represents the study selection process, detailing the number of records that were identified, screened, and excluded at each stage. This structured methodology minimizes selection bias and enhances the reliability of the findings.

### 3.2. Study Characteristics

Table 1 provides a detailed summary of the characteristics of the experimental studies included in this review. Nine experimental studies were analyzed, focusing on various lens materials and coatings that represent advances in the mechanical properties, optical performance, and overall functionality of contact lenses. The materials that were investigated included cellulose-derived hydrogels, hydroxypropyl cellulose hydrogels, pH-sensitive polymers, and advanced composites such as poly(2-hydroxyethyl methacrylate)/β-cyclodextrin-hyaluronan (pHEMA/β-CD-crHA) and silicone materials with enhanced oxygen permeability. Several studies also incorporated cutting-edge technologies, such as self-lubricating lenses and nanogels for controlled drug delivery. The coatings that were employed were specifically designed to optimize biocompatibility, enable sustained drug release, and enhance mechanical strength.

The results reveal notable improvements in key properties. The optical transparency of the studied lenses showed significant enhancement, with many materials demonstrating high light transmission and superior clarity. The drug delivery performance of pH-sensitive polymers and silicone hydrogels was particularly impressive, as they achieved sustained release durations of up to 10 days or more. The biocompatibility was uniformly high across all studies, with materials showing excellent cytocompatibility and minimal toxicity risks. Mechanical properties also saw considerable advancements: hydrogels exhibited improved tensile strength, flexibility, and wear resistance, while elastic moduli were fine-tuned to provide greater functionality and comfort. These findings underscore the significant progress being made in the development of advanced lens materials and coatings.

### 3.3. Outcomes

**Figure 3:**
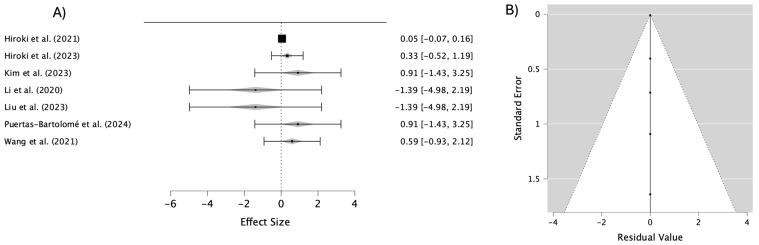
(**A**) Forest plot of transparency in different materials; (**B**) funnel plot to assess publication bias in studies on transparency in different materials [32,33,34,35,36,38,39].

Figure 3 presents the pooled results from the studies that evaluated the transparency of various ophthalmic lens materials, including cellulose-derived hydrogels, pH-sensitive polymers, and zwitterionic nanogels. The overall transparency estimate demonstrated an average effect size of 0.288 (95% CI: −0.028–0.604), indicating a positive trend toward improved transparency across the materials analyzed. The heterogeneity among the studies was minimal (I^2^ = 0%), reflecting consistent outcomes across different material types. This consistency suggests that the intrinsic properties of materials, such as their chemical composition and applied surface treatments, play a pivotal role in achieving similar enhancements in transparency.

Notably, materials incorporating advanced features, such as resistance to lipid and protein deposition, exhibited superior transparency. These findings have significant implications for the development of ophthalmic lenses with enhanced optical properties, paving the way for a broader range of clinical applications.

The accompanying funnel plot reveals a symmetrical distribution of the studies around the pooled estimate, indicating no substantial publication bias. The arrangement of data points within the confidence limits confirms that the standard errors align with the estimated effects, further supporting the reliability and validity of the results. These insights underscore the robustness of the analyses and their relevance to advancing lens material design.

**Figure 4:**
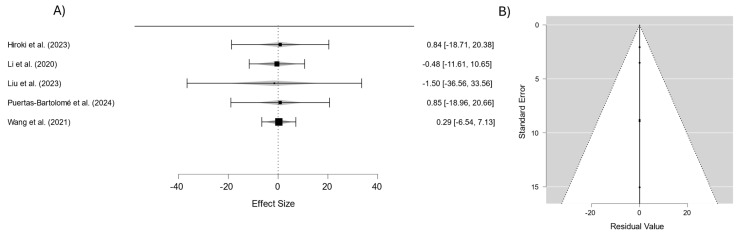
(**A**) Forest plot of water content in different materials; (**B**) funnel plot to assess publication bias in studies on water content in different materials [33,35,36,38,39].

Figure 4 presents the findings of the studies that compared the water content across various contact lens materials. The overall estimated effect size was 0.038 (95% CI: −0.374–0.450), indicating no significant differences in water content among the materials evaluated. The analysis revealed low heterogeneity (I^2^ = 0%), reflecting consistent results irrespective of variations in material composition or manufacturing techniques.

The corresponding funnel plot displays a symmetrical distribution of the studies around the pooled estimate, suggesting an absence of substantial publication bias. Additionally, the majority of data points fall within the confidence limits, demonstrating that the standard errors align proportionally with the observed effect sizes. These results underscore the reliability of the analysis and suggest that the water content properties of modern contact lens materials are largely comparable, offering flexibility in their selection for various clinical and consumer needs.

**Figure 5:**
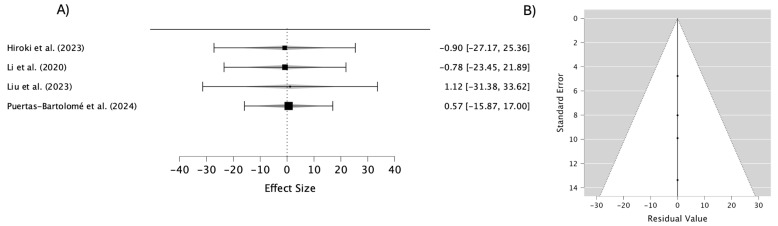
(**A**) Forest plot of oxygen permeability in different materials; (**B**) funnel plot to assess publication bias in studies on oxygen permeability in different materials [33,35,36,38].

Figure 5 depicts the analysis of oxygen permeability across various contact lens materials. The average effect size was 0.034 (95% CI: −0.457–0.524), demonstrating consistent results among the studies analyzed. The low heterogeneity (I^2^ = 0%) indicates that differences in chemical composition or design do not substantially impact the oxygen permeability of the materials that were tested.

The accompanying funnel plot shows a symmetrical distribution of the studies around the average effect line, suggesting no significant publication bias. Additionally, the points are well-contained within the confidence limits, indicating that the standard errors are proportional to the estimated effects. These findings highlight the reliability of the results and suggest that the oxygen permeability remains consistent across different lens materials, regardless of variations in their composition or manufacturing processes.

**Figure 6:**
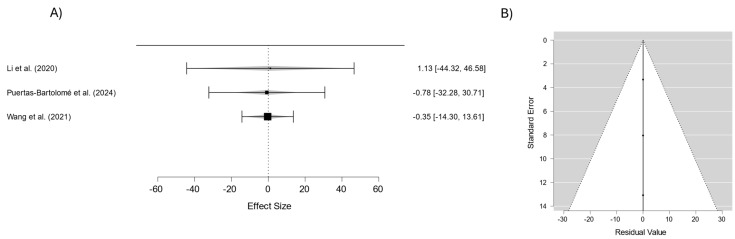
(**A**) Forest plot of the water contact angle in different materials; (**B**) funnel plot to evaluate the publication bias in studies on the water contact angle in different materials [35,38,39].

The combined analysis of the studies that evaluated the water contact angle in contact lens materials (Figure 6) yielded an overall effect estimate of −0.305 (95% CI: −12.589–11.980), indicating no significant differences in this property among the materials analyzed. The low heterogeneity (I^2^ = 0%) highlights the remarkable consistency of the results, suggesting that variations in material composition and design exert minimal influence on the water contact angle behavior. This parameter is crucial for assessing lens wettability, a key determinant of comfort during wear. While the overall findings suggest no substantial differences, individual variations observed in specific materials may hold significance for tailoring lenses to optimize their wettability for diverse user needs.

The associated funnel plot demonstrates a symmetrical distribution of the studies around the mean effect line, indicating no significant publication bias. Additionally, the placement of data points within the confidence limits supports the consistency of the standard errors with the estimated effects. These findings underscore the reliability of the analysis while emphasizing the importance of individual material characteristics in enhancing lens performance.

**Figure 7:**
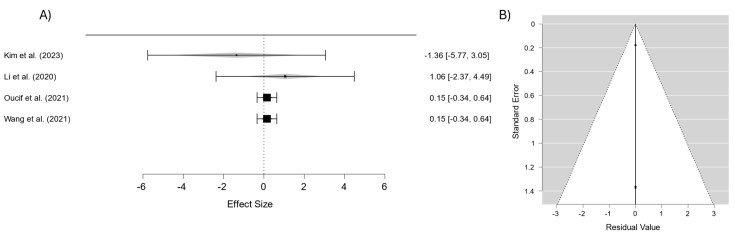
(**A**) Forest plot of cell viability in different materials; (**B**) funnel plot to assess publication bias in studies on cell viability of different materials [34,35,37,39].

The analysis of the studies evaluating the cell viability in contact lens materials (Figure 7) showed an average effect estimate of 0.302 (95% CI: −0.397–1.000), indicating no statistically significant differences in cytocompatibility among the materials analyzed. The absence of heterogeneity (I^2^ = 0%) reflects exceptional consistency across the studies, suggesting that variations in material composition or design did not significantly affect the cell biocompatibility. These findings indicate that the evaluated materials generally exhibit similar properties regarding supporting cell viability.

The corresponding funnel plot reveals a tightly clustered and symmetrical distribution of points around the average effect line, indicating no evidence of significant publication bias. Furthermore, all points fall within the established confidence limits, reinforcing the robustness and reliability of the results. This consistency underscores the high standard of biocompatibility achieved across diverse contact lens materials, offering confidence in their safety and usability.

## 4. Discussion

Publication bias can affect meta-analyses by favoring studies with significant results, but the funnel plots in Figure 3, Figure 4, Figure 5, Figure 6 and Figure 7 suggest this is not a major issue in this study. The symmetrical distribution of data points around the pooled estimates indicates that studies were published regardless of their outcomes. Additionally, the low heterogeneity (I^2^ = 0%) across all figures confirms that the results were consistent across different studies. This strengthens confidence in the findings on the transparency, water content, oxygen permeability, water contact angle, and cell viability of contact lens materials. While subtle biases, such as selective reporting, may still exist, the data overall appear reliable. This suggests that the reported material properties are accurate and applicable in both research and clinical settings.

The results of this meta-analysis highlight significant advancements in the functional properties of materials and treatments used in contact lenses. The included studies demonstrate notable improvements in their optical transparency, biocompatibility, and mechanical properties, underscoring the positive impact of advanced technologies such as cellulose-derived hydrogels and nanogels with sustained release capabilities. These advancements not only enhance the comfort and safety of users but also expand the clinical applications of these materials, such as controlled drug delivery for ocular conditions.

Modern contact lenses are manufactured using advanced materials that optimize their optical transparency, biocompatibility, and mechanical properties. The studies included in this meta-analysis evaluated materials such as cellulose-derived hydrogels, silicone hydrogels, and nanogels with sustained drug release capabilities, all of which have demonstrated improvements in wettability, oxygen permeability, and resistance to protein adhesion. The synthesis of these materials often involves advanced polymer cross-linking techniques and surface modifications, such as plasma treatments and bioinspired coatings. These strategies enhance the mechanical strength, flexibility, and hydration balance of contact lenses, reducing ocular friction and increasing user comfort.

The materials used in contact lens manufacturing directly influence their clinical performance, including their oxygen permeability, moisture retention, and adaptability to the cornea. Among the most widely used materials in the market are Comfilcon A, a silicone hydrogel with high oxygen permeability that is commonly used in extended-wear lenses that are designed to minimize corneal hypoxia; Lotrafilcon B, a silicone hydrogel with a plasma surface treatment that reduces lipid deposition and enhances its durability; and Nelfilcon A, a high-water-content hydrogel that is primarily used in daily disposable lenses, for which hydration and comfort are prioritized. The findings of this meta-analysis suggest that the oxygen permeability remains consistent across different material compositions, reinforcing the importance of surface treatments in improving comfort and resistance to protein deposits.

Advancements in contact lens materials and treatments have direct applications in various areas. Increased oxygenation and moisture retention benefit users who are prone to dry eyes and corneal hypoxia, especially those who wear extended-use lenses. The incorporation of materials with controlled drug-release capabilities opens up new possibilities for treating ocular conditions such as glaucoma and dry eye syndrome by reducing the need for multiple daily medication applications. Improved biocompatibility and reduced protein accumulation on the lens surface minimize ocular irritation and enhance tolerance, particularly for individuals with sensitive corneas. Additionally, the environmental sustainability of contact lens materials has become an increasingly relevant concern. The development of biodegradable materials and effective recycling strategies is a crucial step toward reducing the environmental impact of the optical sector.

Compared to prior research, our findings align on the importance of biocompatibility and resistance to protein adsorption as key factors for improving the functionality of contact lenses. The hydrogel materials evaluated in this study demonstrated superior properties compared to traditional hydrogels, consistent with the studies by Liu et al. [6] and Puertas-Bartolomé et al. [38], which emphasized the relevance of self-lubricating treatments and nanogels in reducing friction and increasing comfort. Notably, the oxygen permeability levels remained consistent across the evaluated materials, regardless of their chemical composition, suggesting that advanced treatments can effectively mitigate the intrinsic limitations of certain materials.

However, our findings diverge from those of some previous studies on water content and contact angle measurements [13,22,38] where variability was observed. This discrepancy underscores the need for further optimization of these parameters to improve lens wettability and hydration. Future research could explore targeted modifications of surface treatments to address these gaps.

The clinical implications of these advancements are significant. Improvements in optical transparency and oxygen permeability enhance visual acuity and corneal health, respectively, while biocompatibility reduces the risk of inflammation and discomfort [1,3,20]. For instance, pH-sensitive polymers and silicone hydrogels—which showed sustained drug release capabilities—could revolutionize the management of ocular conditions by offering controlled delivery systems that minimize the need for frequent applications. Notably, the release duration of these materials may differ from that of commercially available drug-delivery lenses, which often rely on diffusion-based mechanisms [1,34,40]. Comparative studies suggest that advanced materials like nanogels and pH-responsive polymers can provide more sustained and tunable release profiles, potentially enhancing the therapeutic efficacy of this method while reducing side effects associated with fluctuating drug concentrations [41,42]. Further research is needed to directly compare these materials with existing commercial formulations to optimize their clinical applications. From a user perspective, the advancements in the mechanical properties of contact lenses, such as increased tensile strength and flexibility, directly translate to greater comfort and adaptability, particularly for individuals with sensitive or irregular corneas. Additionally, the reduced deposition of proteins and lipids on lenses ensures prolonged clarity and reduces irritation risks, offering a more seamless user experience.

Our results also reflect the environmental challenges associated with the widespread use of contact lenses, particularly regarding their disposal. The lack of effective recycling strategies and biodegradable materials for contact lenses remains a critical obstacle. As noted by Rolsky et al. [43], 21% of users dispose of their lenses through drainage systems, significantly contributing to the microplastics issue. This highlights the urgent need for regulated disposal protocols and non-toxic recycling processes that promote a circular economy. Although silicone hydrogels exhibit poor biodegradability due to their hydrophobic nature, they remain a crucial part of this analysis due to their widespread clinical use and superior oxygen permeability [1,23,44]. Emerging research has explored ways to improve their environmental profile, such as incorporating degradable crosslinkers or surface treatments that enhance their microbial degradation [2,3]. Shaker et al. [44] highlight the ability of certain microorganisms and enzymes to degrade polymeric lenses under controlled conditions, offering a potential solution for the sustainable management of ophthalmic waste. Hydrogels, with their greater susceptibility to microbial degradation, provide a promising starting point, while silicones, due to their hydrophobic nature, present a greater challenge in terms of biodegradability. Furthermore, Encarnação et al. [45] propose an innovative model which uses microalgae to treat wastewater contaminated with ophthalmic waste. This approach not only reduces the carbon footprint of contact lenses but also enables biomass recovery for biopolymer production, representing an integrated solution to address the environmental impact of and promote sustainability in the contact lens industry. In this context, it is evident that balancing technological innovation with environmental sustainability is essential for the future development of the industry. The integration of biodegradable materials alongside effective recycling technologies would be a vital step toward reducing the environmental impact of the contact lens industry. At the same time, regulatory policies must play an active role in incentivizing and enforcing sustainable practices throughout the product life cycle.

Another critical aspect of sustainability in the ophthalmic sector is the issue of lens recycling. Oliveira et al. [46] found that 40.8% of surveyed Portuguese optical centers do not engage in any form of recycling for optical materials, which reflects a significant gap in environmental stewardship within the sector. For the ophthalmic lens industry to transition toward greater sustainability, it is essential to invest in continued research and innovation. This includes developing biodegradable and eco-friendly materials that meet the industry’s stringent standards for quality and durability, as well as implementing more effective recycling practices to reduce waste.

Among the limitations of this study, the methodological heterogeneity of the included studies and the limited availability of longitudinal data on the impact of the studied materials in long-term contact lens use stand out. Additionally, although random-effects models were employed to address variability between studies, further research analyzing the performance of these materials under real-world conditions would be beneficial. Another limitation is the sample size, as only nine studies were included in the meta-analysis. However, this was a direct result of the strict inclusion criteria, which ensured that only studies with comparable methodologies and outcome measures were analyzed. This approach minimizes the methodological inconsistencies and enhances the reliability of the pooled results. Furthermore, the low heterogeneity (I^2^ = 0%) across all figures supports the coherence and robustness of the findings, suggesting that the selected studies provide valid and consistent data. Although 27 studies were excluded due to methodological inconsistencies, incomplete data, or a high risk of bias, their exclusion does not appear to impact the overall conclusions, as the included studies maintain methodological rigor and comparability. While this limitation is acknowledged, the findings of this study contribute valuable insights regarding contact lens materials, and future research should aim to incorporate larger, multicenter trials and longitudinal studies to further validate these results and improve their generalizability.

Future research could focus on exploring combinations of materials and treatments that simultaneously optimize the functionality, biocompatibility, and sustainability of contact lenses. The integration of advanced polymers, nanomaterials, and bioengineered coatings could further enhance the oxygen permeability, moisture retention, and antimicrobial properties of contact lenses, addressing key challenges in long-term wearability and patient comfort. Additionally, the development of smart contact lenses with integrated biosensors and drug-delivery systems represents a promising avenue for real-time health monitoring and precision medicine. These lenses could track ocular and systemic biomarkers, enabling the early detection and management of conditions such as diabetes, glaucoma, and dry eye syndrome. Future efforts should focus on optimizing the energy efficiency, wireless communication, and safety of these wearable ophthalmic devices to facilitate their clinical adoption. From an environmental perspective, continued research into biodegradable and recyclable materials to minimize the ecological footprint of disposable lenses is essential. Investigating enzymatically degradable polymers, bio-based alternatives, and sustainable production methods could lead to more eco-friendly solutions in the optical industry. This meta-analysis establishes a solid foundation which can guide future research and fosters a holistic vision that integrates scientific advancements, clinical needs, and environmental considerations. A multidisciplinary approach that combines materials science, biomedical engineering, and regulatory developments will be crucial in shaping the next generation of contact lenses.

## 5. Conclusions

Contact lenses exemplify the intersection of science, technology, and healthcare. From their historical roots to their current multifunctional applications, they continue to evolve, improving the quality of life for millions while addressing diverse ocular and systemic health needs. As innovation accelerates, contact lenses are set to play an even more significant role in personalized and sustainable eye care.

The chemistry underlying contact lens materials is critical to their functionality, comfort, and sustainability. Advances in polymer science have enabled the achievement of an optimal balance between the oxygen permeability, hydration, and mechanical strength of contact lenses. Future research will likely focus on developing eco-friendly materials and recycling technologies to reduce the environmental impact of disposable lenses. Additionally, studies may explore optimizing material combinations to enhance both the performance and sustainability of contact lenses, while the development of smart contact lenses with health-monitoring features could transform the management of ocular and systemic conditions.

## Figures and Tables

**Table 1:** Baseline characteristics of studies on sustainable materials for contact lenses.

Study	Year	Lens Material/Coating	Key Findings	Drug Release Characteristics	Mechanical Properties
**Hiroki et al. (2021)** [32]	2021	Cellulose derivative-based hydrogels	Improved mechanical properties and elasticity	Not applicable	Tensile strength 0.2 MPa
**Hiroki et al. (2023)** [33]	2023	Hydroxypropyl cellulose hydrogels	Biodegradable, high transparency, reduced protein deposition	Sustained release for >48 h	Tensile strength 0.2 MPa
**Kim et al. (2023)** [34]	2023	pH-sensitive polymer with silica	Controlled, temperature-sensitive drug release	pH-sensitive release for <35 °C	Elastic modulus maintained
**Li et al. (2020)** [35]	2020	pHEMA/β-CD-crHA hydrogels *	Improved wettability and sustained drug delivery	Prolonged release for 72 h	Elastic modulus 1.8 MPa
**Liu et al. (2023)** [36]	2023	Silicone hydrogels	High oxygen permeability and controlled drug release	Release improved by 200%	Oxygen permeability 65.8
**Oucif et al. (2021)** [37]	2021	HEMA-co-HEA hydrogels **	Adjustable swelling and extended drug release	Non-Fickian release behavior	Enhanced swelling properties
**Puertas-Bartolomé et al. (2024)** [38]	2024	Self-lubricating hydrogel lenses	Long-term lubrication and HA secretion	Sustained HA *** release over 3 weeks	Functional flexibility
**Wang et al. (2021)** [39]	2021	Nanogel-embedded lenses	Extended drug release and improved bioavailability	Sustained drug delivery for 10 days	Mechanical durability maintained

* poly(2-hydroxyethyl methacrylate)/β-cyclodextrin-hyaluronan (pHEMA/β-CD-crHA); ** poly(hydroxyethyl methacrylate-co-hydroxyethyl acrylate) (HEMA-co-HEA); *** hyaluronan.

## Data Availability

The original contributions presented in this study are included in the article/Supplementary Materials. Further inquiries can be directed to the corresponding author.

## Supplementary Materials

The following supporting information can be downloaded at: https://www.mdpi.com/article/10.3390/ma18071445/s1, Supplementary Materials S1: Risk of Bias Assessment, Bibliographic Search Strategy, and PRISMA 2020 Checklist.
