# Supplementary material for: Meta-Analysis of Materials and Treatments Used in Contact Lenses: Implications for Lens Characteristics
Source: Materials (Basel). 2025 Mar 25;18(7):1445. doi: 10.3390/ma18071445 (PMC11989920; doi:10.3390/ma18071445)
Supplement: Supplementary file 1 [file materials-18-01445-s001.zip › materials-3496942-supplementary.pdf]

Supplementary File S1. Risk of Bias Assessment, Bibliographic Search Strategy, and PRISMA 2020 Checklist

RISK OF BIAS JUDGEMENT

1. Hiroki et al. (2021)

Criterion	Evaluation	Justification
**Bias due to selection**	Low	The study explicitly outlines the criteria for material selection, the sources of reagents, and the methods used for irradiation.
**Bias due to performance**	Low	Well-documented procedures were followed to evaluate the mechanical, chemical, and optical properties of the hydrogels.
**Bias due to detection**	Low	The methods used, such as spectrophotometry and tensile testing, are adequately described and appropriate for the study's objectives.
**Bias due to reproducibility**	Low	The authors clearly describe the preparation of the hydrogels, the equipment used for measurements, and the specific conditions under which the experiments were conducted. This includes detailed protocols for transparency measurements, mechanical testing, and biodegradability evaluations. Additionally, all reagents and their sources are listed, enhancing the reproducibility of the work.
**Bias due to reporting**	Low	The article provides a balanced presentation of both positive findings and limitations of the studied materials.

2. Hiroki et al. (2023)

Criterion	Evaluation	Justification
**Bias due to selection**	Low	The study explicitly outlines the selection criteria for materials, including sources and preparation protocols, reducing the likelihood of selection bias.
**Bias due to performance**	Low	Detailed protocols for hydrogel preparation, irradiation, and testing of mechanical and optical properties were consistently followed, ensuring reliable performance outcomes.
**Bias due to detection**	Low	The study employed validated techniques, including spectrophotometry for transparency and tensile testing for mechanical properties, ensuring accuracy and consistency in data collection.
**Bias due to reproducibility**	Low	Comprehensive methodological descriptions, including hydrogel composition, preparation methods, and testing conditions, enhance the reproducibility of the results.
**Bias due to reporting**	Low	The article presents both the strengths and limitations of the materials studied, offering a balanced and transparent account of the findings.

3. Kim et al. (2023)

Criterion	Evaluation	Justification
**Bias due to selection**	Low	The preparation of the pH-sensitive contact lenses and their incorporation with mesoporous silica nanoparticles (MSNs) is thoroughly explained

**Bias due to performance**	Low	The controlled pH and temperature environments in both in vitro drug release and cytotoxicity tests were appropriately maintained and documented.
**Bias due to detection**	Low	Techniques such as UV-Vis spectroscopy for drug quantification and atomic force microscopy for surface roughness were carefully documented and validated.
**Bias due to reproducibility**	Low	Detailed protocols for polymerization, drug incorporation, and experimental setups ensure the study can be replicated by other researchers.
**Bias due to reporting**	Low	The study provides comprehensive results, including data on optical properties, drug release profiles, and cytotoxicity, ensuring transparency.

4. Li et al. (2020)

**Criterion**	**Evaluation**	**Justification**
**Bias due to selection**	Low	The study thoroughly explains the preparation of pHEMA/β-CD-crHA hydrogels, including material sourcing and synthesis processes.
**Bias due to performance**	Low	Consistent methodologies for evaluating mechanical, optical, and drug delivery properties were applied across all hydrogel variations.
**Bias due to detection**	Low	Techniques such as spectrophotometry, SEM, and AFM were used, with detailed protocols ensuring accurate and reproducible measurements.
**Bias due to reproducibility**	Low	Comprehensive descriptions of hydrogel synthesis, characterization methods, and testing protocols enhance reproducibility.
**Bias due to reporting**	Low	The article presents detailed findings on hydrophilicity, protein resistance, drug delivery efficiency, and cytotoxicity, ensuring transparency.

5. Liu et al. (2023)

**Criterion**	**Evaluation**	**Justification**
**Bias due to selection**	Low	The preparation and optimization of silicone hydrogel formulations were described in detail, including material sources and synthesis protocols.
**Bias due to performance**	Low	The controlled polymerization techniques and defined environmental conditions (e.g., temperature and humidity) were maintained throughout the study.
**Bias due to detection**	Low	Key evaluations such as oxygen permeability, wettability, and transparency were conducted using well-documented and reliable methods, including spectrophotometry and isobaric techniques.
**Bias due to reproducibility**	Low	The comprehensive description of materials, preparation methods, and analysis techniques ensures reproducibility. The use of Gaussian09W for molecular simulation further enhances transparency.
**Bias due to reporting**	Low	Results are presented comprehensively, including both primary findings and limitations. Data on drug release, hydrophilicity, and mechanical properties are detailed and well-documented.

6. Oucif et al. (2021)

Criterion	Evaluation	Justification
**Bias due to selection**	Low	Selection of monomers, cross-linkers, and other reagents was clearly described, ensuring replicability.
**Bias due to performance**	Low	Consistent environmental conditions (temperature, pH, and saline solution preparation) and defined testing methodologies were applied.
**Bias due to detection**	Low	The study utilized established and reliable analytical methods for data collection and analysis.
**Bias due to reproducibility**	Low	Steps for hydrogel synthesis and characterization were described with precision, including conditions for copolymerization and cross-linking.
**Bias due to reporting**	Low	Transparency in reporting was demonstrated, with clear descriptions of experimental procedures and data interpretation.

7. Puertas-Bartolomé et al. (2024)

Criterion	Evaluation	Justification
**Bias due to selection**	Low	The study explicitly outlines the design of the hydrogel matrix and the embedded biofactories, including material composition and preparation protocols.
**Bias due to performance**	Low	Procedures for hydrogel synthesis, bacterial growth, and property testing were consistent and followed standardized methods.
**Bias due to detection**	Low	The study employs advanced techniques such as confocal microscopy, rheology, and spectrophotometry to ensure accurate data collection.
**Bias due to reproducibility**	Low	Comprehensive descriptions of hydrogel preparation, bacterial encapsulation, and functional testing support reproducibility.
**Bias due to reporting**	Low	The article presents a transparent account of the results, including supplementary data for key findings.

8. Wang et al. (2021)

Criterion	Evaluation	Justification
**Bias due to selection**	Low	Detailed methods for selecting zwitterionic nanogels, monomers, and cross-linkers are included, ensuring a systematic approach.
**Bias due to performance**	Low	Comprehensive descriptions of preparation methods, such as polymerization and drug loading, ensure uniformity in testing conditions.
**Bias due to detection**	Low	Techniques such as UV-Vis spectroscopy, SEM, and FTIR analysis were properly calibrated and employed.
**Bias due to reproducibility**	Low	Protocols for drug loading, nanogel synthesis, and contact lens preparation are provided step by step, enhancing reproducibility.
**Bias due to reporting**	Low	The study transparently discusses outcomes such as drug release profiles, hydrophilicity, and cytocompatibility, with appropriate visual aids and statistical analyses.

PUBMED SEARCH STRATEGY

Search: **(Sustain* OR Recycled material*) AND "Contact lens*"**
("sustain*"[All Fields] OR (("recyclability"[All Fields] OR "recyclable"[All Fields] OR "recyclables"[All Fields] OR "recyclate"[All Fields] OR "recyclates"[All Fields] OR "recycler"[All Fields] OR "recyclers"[All Fields] OR "recycles"[All Fields] OR "recycling"[MeSH Terms] OR "recycling"[All Fields] OR "recycle"[All Fields] OR "recycled"[All Fields] OR "recyclings"[All Fields]) AND "material*"[All Fields])) AND "contact lens*"[All Fields]

Translations

Recycled: "recyclability"[All Fields] OR "recyclable"[All Fields] OR "recyclables"[All Fields] OR "recyclate"[All Fields] OR "recyclates"[All Fields] OR "recycler"[All Fields] OR "recyclers"[All Fields] OR "recycles"[All Fields] OR "recycling"[MeSH Terms] OR "recycling"[All Fields] OR "recycle"[All Fields] OR "recycled"[All Fields] OR "recyclings"[All Fields]

PRISMA 2020 Checklist

Section and Topic	Item #	Checklist item	Location where item is reported
**TITLE**			
Title	1	Identify the report as a systematic review.	1
**ABSTRACT**			
Abstract	2	See the PRISMA 2020 for Abstracts checklist.	1
**INTRODUCTION**			
Rationale	3	Describe the rationale for the review in the context of existing knowledge.	2
Objectives	4	Provide an explicit statement of the objective(s) or question(s) the review addresses.	3
**METHODS**			
Eligibility criteria	5	Specify the inclusion and exclusion criteria for the review and how studies were grouped for the syntheses.	3
Information sources	6	Specify all databases, registers, websites, organisations, reference lists and other sources searched or consulted to identify studies. Specify the date when each source was last searched or consulted.	3
Search strategy	7	Present the full search strategies for all databases, registers and websites, including any filters and limits used.	4
Selection process	8	Specify the methods used to decide whether a study met the inclusion criteria of the review, including how many reviewers screened each record and each report retrieved, whether they worked independently, and if applicable, details of automation tools used in the process.	4
Data collection process	9	Specify the methods used to collect data from reports, including how many reviewers collected data from each report, whether they worked independently, any processes for obtaining or confirming data from study investigators, and if applicable, details of automation tools used in the process.	4
Data items	10a	List and define all outcomes for which data were sought. Specify whether all results that were compatible with each outcome domain in each study were sought (e.g. for all measures, time points, analyses), and if not, the methods used to decide which results to collect.	4
	10b	List and define all other variables for which data were sought (e.g. participant and intervention characteristics, funding sources). Describe any assumptions made about any missing or unclear information.	4
Study risk of bias assessment	11	Specify the methods used to assess risk of bias in the included studies, including details of the tool(s) used, how many reviewers assessed each study and whether they worked independently, and if applicable, details of automation tools used in the process.	4
Effect measures	12	Specify for each outcome the effect measure(s) (e.g. risk ratio, mean difference) used in the synthesis or presentation of results.	5
Synthesis methods	13a	Describe the processes used to decide which studies were eligible for each synthesis (e.g. tabulating the study intervention characteristics and comparing against the planned groups for each synthesis (item #5)).	5
	13b	Describe any methods required to prepare the data for presentation or synthesis, such as handling of missing summary statistics, or data conversions.	5
	13c	Describe any methods used to tabulate or visually display results of individual studies and syntheses.	5
	13d	Describe any methods used to synthesize results and provide a rationale for the choice(s). If meta-analysis was performed, describe the model(s), method(s) to identify the presence and extent of statistical heterogeneity, and software package(s) used.	5
	13e	Describe any methods used to explore possible causes of heterogeneity among study results (e.g. subgroup analysis, meta-regression).	5
	13f	Describe any sensitivity analyses conducted to assess robustness of the synthesized results.	5
Reporting bias assessment	14	Describe any methods used to assess risk of bias due to missing results in a synthesis (arising from reporting biases).	5
Certainty assessment	15	Describe any methods used to assess certainty (or confidence) in the body of evidence for an outcome.	5

PRISMA 2020 Checklist

Section and Topic	Item #	Checklist item	Location where item is reported
**RESULTS**			
Study selection	16a	Describe the results of the search and selection process, from the number of records identified in the search to the number of studies included in the review, ideally using a flow diagram.	6-11
	16b	Cite studies that might appear to meet the inclusion criteria, but which were excluded, and explain why they were excluded.	6-11
Study characteristics	17	Cite each included study and present its characteristics.	6-11
Risk of bias in studies	18	Present assessments of risk of bias for each included study.	5
Results of individual studies	19	For all outcomes, present, for each study: (a) summary statistics for each group (where appropriate) and (b) an effect estimate and its precision (e.g. confidence/credible interval), ideally using structured tables or plots.	6-11
Results of syntheses	20a	For each synthesis, briefly summarise the characteristics and risk of bias among contributing studies.	6-11
	20b	Present results of all statistical syntheses conducted. If meta-analysis was done, present for each the summary estimate and its precision (e.g. confidence/credible interval) and measures of statistical heterogeneity. If comparing groups, describe the direction of the effect.	6-11
	20c	Present results of all investigations of possible causes of heterogeneity among study results.	6-11
	20d	Present results of all sensitivity analyses conducted to assess the robustness of the synthesized results.	-
Reporting biases	21	Present assessments of risk of bias due to missing results (arising from reporting biases) for each synthesis assessed.	-
Certainty of evidence	22	Present assessments of certainty (or confidence) in the body of evidence for each outcome assessed.	-
**DISCUSSION**			
Discussion	23a	Provide a general interpretation of the results in the context of other evidence.	11-12
	23b	Discuss any limitations of the evidence included in the review.	11-12
	23c	Discuss any limitations of the review processes used.	11-12
	23d	Discuss implications of the results for practice, policy, and future research.	11-12
**OTHER INFORMATION**			
Registration and protocol	24a	Provide registration information for the review, including register name and registration number, or state that the review was not registered.	3
	24b	Indicate where the review protocol can be accessed, or state that a protocol was not prepared.	3
	24c	Describe and explain any amendments to information provided at registration or in the protocol.	3
Support	25	Describe sources of financial or non-financial support for the review, and the role of the funders or sponsors in the review.	13
Competing interests	26	Declare any competing interests of review authors.	13
Availability of data, code and other materials	27	Report which of the following are publicly available and where they can be found: template data collection forms; data extracted from included studies; data used for all analyses; analytic code; any other materials used in the review.	-

From: Page MJ, McKenzie JE, Bossuyt PM, Boutron I, Hoffmann TC, Mulrow CD, et al. The PRISMA 2020 statement: an updated guideline for reporting systematic reviews. BMJ 2021;372:n71. doi: 10.1136/bmj.n71. This work is licensed under CC BY 4.0. To view a copy of this license, visit https://creativecommons.org/licenses/by/4.0/
